# Sulfur hexafluoride (SF_6_) versus perfluoropropane (C_3_F_8_) tamponade and short term face-down position for macular hole repair: a randomized prospective study

**DOI:** 10.1186/s40942-016-0036-9

**Published:** 2016-04-01

**Authors:** Giamberto Casini, Pasquale Loiudice, Stefano De Cillà, Paolo Radice, Marco Nardi

**Affiliations:** 1 Ophthalmology Unit, Department of Surgical, Medical, Molecular and Critical Area Pathology, University of Pisa, Via Paradisa 2, 56124 Pisa, Italy; 2 Eye Clinic, San Paolo Hospital, University of Milano, Milano, Italy; 3 Ospedale Fatebenefratelli e Oftalmico, Milano, Italy

**Keywords:** Gas tamponade, Inner limiting membrane peeling, Macular hole volume, Perfluoropropane (C_3_F_8_), Sulfur hexafluoride (SF_6_), Vitrectomy

## Abstract

**Background:**

To compare early visual and anatomical outcomes after either sulfur hexafluoride (SF_6_) or perfluoropropane (C_3_F_8_) tamponade for macular hole repair.

**Methods:**

147 eyes affected by primary full-thickness macular hole underwent pars plana vitrectomy with dye assisted removal of the internal limiting membrane and gas tamponade. Prone position was prescribed for 48 h after surgery. All patients were divided into 3 groups depending on the size of the hole: small (<250 µm), medium (>250–<400 µm) or large (>400 µm). Eyes within the same group randomly received either SF_6_ (70 eyes) or C_3_F_8_ (77 eyes). A complete ophthalmic evaluation, including best corrected visual acuity and anatomic status of the macular holes, was conducted preoperatively, at 1 week and 1 month after surgery. Macular hole volume was calculated using optical coherence tomography scans. The Wilcoxon Signed Ranks Test, the Mann–Whitney Test, the Spearman’s rank-order correlation coefficient and the study of variance for repeated measures were used for statistical analysis.

**Results:**

Mean best-corrected visual acuity improved from 0.92 logMAR to 0.28 logMAR (P < 0.001). A reduction of the dimensions of macular holes was observed in all cases, with a total repair of 90 % (63/70 eyes) in the SF_6_ group and 91 % in the C_3_F_8_ group (70/77 eyes). There was a negative correlation between the initial minor diameter, the volume of the hole and the rate of anatomic success.

**Conclusions:**

Short-term anatomical and visual outcomes were similar in eyes treated with either SF_6_ or C_3_F_8_, independently of the stage of the macular hole. The initial volume and the minor diameter of the hole may be considered as valid tools for predicting surgical success. Age and gender did not appear to have influenced the prognosis.

## Background

A macular hole (MH) is a full thickness defect of the retinal neuroepithelium involving the fovea [1]. It is idiopathic in most cases and is the result of a centrifuge movement of photoreceptors, under the effect of vitreomacular forces [2]. Idiopathic macular hole (IMH) has an incidence rate from 7.8 to 30 cases every 100,000 citizens [3] and it is three times more frequent in women [4]. IMH usually affects only one eye, but it can be found in both eyes in 11.7 % of the cases. The risk for a patient with macular hole in one eye, to develop it in the fellow one, ranges from 2 to 15 %, depending on the presence of vitreous detachment [5]. Female gender and age over 65 years are present in 67–72 % of the cases [6].

The most widely accepted hypothesis concerning idiopathic macular hole pathogenesis is an abnormal anteroposterior vitreous traction [7]. There is an adhesive interaction between the posterior vitreous cortex and the internal limiting membrane (ILM). When age-related physiologic posterior vitreous detachment occurs, vitreous cortex remnants persist on the retina surface in 44 % of eyes and may form a layer [8]. An anomalous foveal vitreoretinal adherence with perifoveal vitreous detachment may be associated to vitreomacular traction, leading to distortion of the foveal surface and hole formation. Furthermore, fibrocellular proliferation on the top of the inner retinal surface is supposed to support the formation of macular holes. Fibrocellular proliferation is also suspected to be responsible for the reopening of the macular hole or the persistence of the hole in spite of surgery [9]. Gass and Johnson [10, 11] described a classification scheme for idiopathic macular holes and their prodromal injuries. In 1995, Gass [7] proposed a microscopic classification of stages of development of a macular hole. More recently the International Vitreomacular Traction Study (IVTS) Group developed an optical coherence tomography (OCT)-based anatomic classification system for diseases of the vitreomacular interface [12]. It has been clinically established that impending macular holes have a 50 % chance to evolve to a spontaneous closure with the resolution of symptoms [6]. They are therefore observed and not surgically treated. However, a spontaneous resolution, with hole closure and a restoration of the normal foveal contour, is very rare in full-thickness macular holes. It occurs in 2–4 % of the eyes [13, 14] therefore these cases are usually treated surgically by pars plana vitrectomy, with or without peeling of the internal limiting membrane.

Kelly and Wendel pioneered macular hole surgery in 1991 when they proposed vitrectomy by pars plana (PPV) with gas tamponade [15]. Internal limiting membrane peeling can be associated to vitrectomy in order to remove any tractional component.

Peeling of ILM has a success ranging from 92 to 97 % whereas PPV without peeling has success ranging from 78 to 89 % [16–20]. Biomaterials are used to replace the vitreous offering the advantage to stably buffer the retina, reduce intraocular streams, sustain globe volume and favour the flattening of the retinal profile. In macular hole surgery, gas tamponade represents the first choice among biomaterials, including sterile air, perfluoropropane and sulfur hexafluoride, which are the most frequently used.

## Methods

Between November 2014 and July 2015, 147 eyes of 141 patients affected by small (<250 µm), medium (>250–<400 µm) or large (>400 µm) idiopathic full-thickness macular holes were assessed for this study at the Department of Ophthalmology, Pisa University, Italy. We received approval by the Ethical Review Board of the University of Pisa. The study was performed in adherence to the tenets of the Declaration of Helsinki; all patients signed an informed consent form.

To be eligible, patients had to show a macular hole diagnosis confirmed by fundus examination and optical coherence tomography (OCT) images.

Preoperatively, at 1 week and 1 month after surgery, all the patients underwent a complete ophthalmic examination, including best corrected visual acuity (BCVA), Goldman applanation tonometry, fundus examination and optical coherence tomography (OCT) (3D OCT-2000 Spectral Domain, Topcon, Japan) using Macular Mode tool of the instrument (3D 6.0 × 6.0 mm; 512 × 128). The surgical treatment included 25 Gauge pars plana vitrectomy (PPV), posterior vitreous detachment, peeling of the epiretinal membrane, dye assisted peeling of the internal limiting membrane (ILM) using brilliant blue G (Brilliant Peel^®^, Fluoron GmbH, Ulm, Germany) and an injection of gas tamponade. The stained ILM was peeled using an end gripping forceps (Grieshaber Asymmetrical Forceps, DSP, Alcon, Fort Worth, Texas, USA). Gas fluid exchange was performed with either 20 % sulfur hexafluoride (SF_6_, ALA SF6—111201, Alamedics GMBH & CO. KG) or 14 % perfluoropropane (C_3_F_8_, ALA C3F8—111401, Alamedics GMBH & CO. KG). All patients laid in prone position for 48 h after surgery. Phakic patients also underwent cataract surgery in the same time as the vitrectomy. Patients with secondary macular holes, previous retinal detachment and recurrence holes were excluded from the study.

We divided all the patients into three groups: small (<250 µm), medium (>250–<400 µm) and large (>400 µm) macular holes according to the International Vitreomacular Traction Study Group Classification [12]. The size of the macular hole was defined as the minimum hole width drawing a line with the caliper parallel to the retinal pigment epithelium. Patients within the same group were randomly treated either with SF_6_ or C_3_F_8_.

**Fig. 1:**
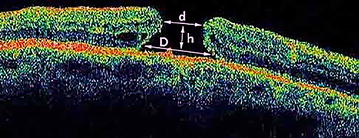
The shape of the hole approximately resembles a truncated cone. To calculate the volume (V) of the macula hole we used the minor diameter (d), the major diameter (D) and the height (H) of the hole

OCT scans let us calculate the volume (V) of the macula hole using the minor diameter (d), the major diameter (D) and the height (H) of the hole. The shape of the hole approximately resembles a truncated cone (Fig. 1): in this case, the volume can be calculated using the following formula

$$V = \frac{1}{3}\pi h\left[ {\left( {\frac{D}{2}} \right)^{2} + \left( {\frac{D}{2}*\frac{d}{2}} \right)^{2} + \left( {\frac{d}{2}} \right)^{2} } \right]$$

Statistical analysis was performed using counts and percentages for qualitative variables (gender or type of tamponade) and ordinal variables (stage); media and standard deviation for quantitative Gaussian variables (age); medians and interquartile ranges for non-Gaussian quantitative variables (volume of macular hole). A contingency table was used to display the frequency distribution of type of the gas used and stage of the macular hole. Spearman’s test was used to analyse correlation between the two variables. The trend of visual acuity over time was graphically displayed using lines charts. We used Wilcoxon Signed Ranks Test to analyse modification of visual acuity and hole volume over time. The rate of improvement at each time point was compared in each group using the Mann–Whitney Test. The Spearman’s rank-order correlation coefficient test was used to analyse correlation between initial major diameter, minor diameter and volume of the macular hole and rate of anatomic success. Inferential analysis was made with the study of variance for repeated measures. We also evaluated some possible bias such as age and gender. Statistical analysis was completed using software SPSS (Ver 21.0) for windows. Differences were considered significant when *P* < 0.05.

## Results

147 eyes of 141 patients, with a mean age of 72 ± 9 years, were included in the study. 70 of them underwent PPV and SF_6_ tamponade; 77 patients PPV and C_3_F_8_ tamponade. According to IVTS classification [12], 47 patients presented a macular hole classified as small, 51 as medium and 49 as large. We divided all the patients into three groups, depending on the stage of the macular hole. Patients within the same group were randomly treated either with SF_6_ or C_3_F_8_. The distribution of cases depending on stage and gas tamponade is shown in Table 1.

**Table 1:** Distribution of cases depending on stage and gas tamponade

Type of tamponade	Size of the macular hole			
	Small	Medium	Large	Total
SF6	22	27	21	70
C3F8	25	24	28	77
Total	47	51	49	147

All the patients were divided into three groups depending on the narrowest width of the hole drawing a line with the caliper tool parallel to the retinal pigment epithelium: small (<250 µm), medium (>250–<400 µm) and large (>400 µm). SF_6_ = sulfur hexafluoride; C_3_F_8_ = perfluoropropane

**Fig. 2:**
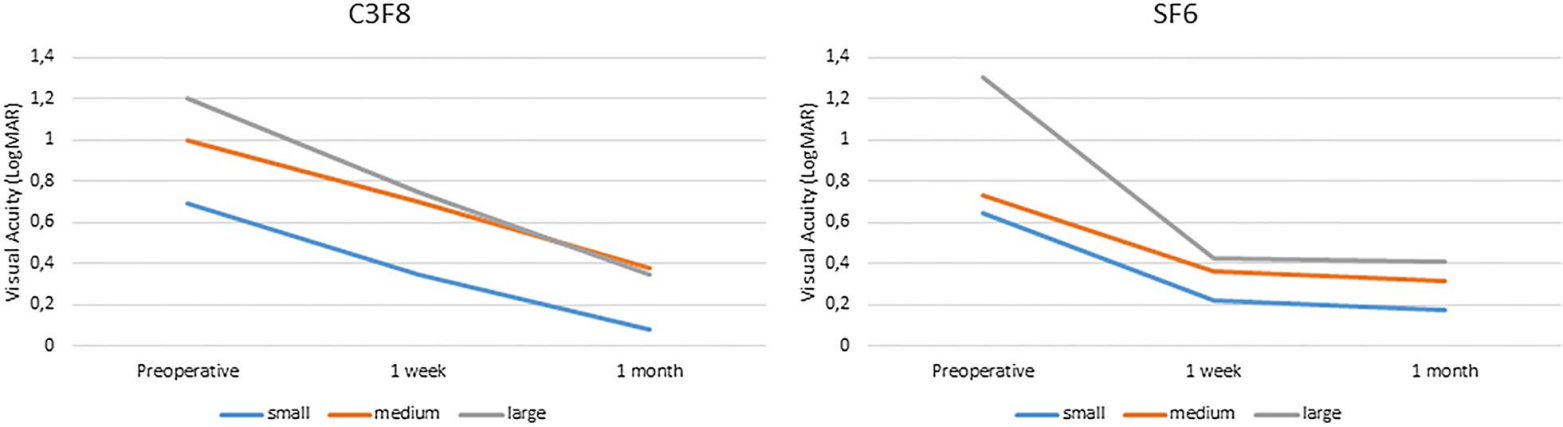
Trend of mean best corrected visual acuity over time, depending on the size of the macular hole and the tamponade used. SF_6_ = sulfur hexafluoride; C_3_F_8_ = perfluoropropane; small (<250 µm), medium (>250–<400 µm) and large (>400 µm)

There was a statistically significant increase in BCVA after surgery in all the three groups with a mean BCVA improvement from 0.92 logMAR to 0.28 logMAR at the last follow up (P < 0.01). Pre and post-operative BCVA measurements are summarized in Table 2. The trend of mean BCVA over time, depending on the stage of the macular hole, is represented in Fig. 2.

**Table 2:** Pre and post-operative values of best corrected visual acuity (BCVA) and macular hole volume (MHV)

Size of the hole	SF6			C3F8		
	Preoperatively	1 week	1 month	preoperatively	1 week	1 month
Small						
BCVA	0.643	0.225	0.175	0.695	0.35	0.085
MHV	21476439.05	2659017.94	125964	22394832.6	2237618.05	117937.53
Medium						
BCVA	0.7333	0.36	0.316	1	0.7	0.38
MHV	38628154.71	1855745.73	1535126.93	37941937.36	1946731.8	1694831.07
Large						
BCVA	1.305	0.426	0.413	1.2	0.75	0.35
MHV	173154648.9	91131802.78	8354755.33	169315684.3	87531967.08	7682391.04

We divided the patients into two groups depending on the type of tamponade used. Visual acuity is expressed in LogMAR and hole volume in µm^3^. SF_6_ = sulfur hexafluoride; C_3_F_8_ = perfluoropropane; small (<250 µm), medium (>250–<400 µm), large (>400 µm)

The rate of improvement at each time point was compared between groups using the Mann–Whitney Test. Patients treated with SF_6_ experienced a greater improvement of their BCVA than those treated with C_3_F_8_ at 1 week postoperatively (61 vs. 39 %) (P < 0.01) independently of the stage of the hole. The difference is not significant at 1 month follow up (66 vs. 74 %). The OCT scans let us calculate major diameter, minor diameter, height and volume of the macular holes.

A reduction of the dimensions of macular holes was observed in all cases and a total repair was obtained in 133 of 147 eyes (90 %). Mean macular hole volumes in each group, at the different time points are displayed in Table 2.

A contingency table (Table 3) shows the number and the percentage of complete closure depending on the stage of disease and the type of gas used. The percentage of anatomic success reduces from small hole group to large hole group (Spearman test, P = 0.009).

**Table 3:** Macular hole closure rate at 1 month postoperatively

Type of tamponade	Size of the macular hole			
	Small	Medium	Large	Total
SF6	21 (95 %)	25 (93 %)	17 (81 %)	63 (90 %)
C3F8	25 (100 %)	22 (92 %)	23 (82 %)	70 (91 %)
Total	46 (98 %)	47 (92 %)	40 (82 %)	133 (90 %)

Number and percentage of complete closure of the macular hole depending on stage and type of tamponade

SF_6_ = sulfur hexafluoride; C_3_F_8_ = perfluoropropane; small (<250 µm), medium (>250–<400 µm), large (>400 µm)

There was a negative correlation between the initial major diameter and the volume of the hole and rate of anatomic success (Spearman’s rank-order correlation coefficient, r_s_ = −0.7, P < 0.05). No correlation was found between the rate of complete anatomic closure and the minor diameter of the hole. No post-operative complications were observed.

## Discussion

The efficacy of pars plana vitrectomy (PPV) for the treatment of macular hole has been known since 1991 when Kelly and Wendel [15] described a five steps technique and reported a success rate of 58 %. Since then, considerable strides forward have been made and the PPV for the treatment of macular hole has become a safe procedure performed worldwide. Recent publications described a rate of anatomic repair that ranges from 80 to 97.1 % [21–24]. However, several topics are still debated, among which whether or not to peel the internal limiting membrane (ILM), the use and the choice of the dye for better IML visualization, whether to prescribe or not the face-down position, its duration and the choice of the tamponade. Usually gas tamponades are preferred even if good results have been reported after heavy silicone oil use [25].

Intraocular gases have the capacity for isolating and sealing to area of the hole; in addition they effect a mechanical tamponade due to the buoyancy of the gas and provide a template for glial cells migration, promoting the healing of the hole.

SF_6_, C_3_F_8_ and sterile air are the most used gas tamponade for macular hole surgery.

Which is the tamponade that ensures the best results is still a debated topic. Kelly and Wendel in their pioneering study used SF_6_, a short acting gas employed in many other subsequent studies. Other authors used a long acting gas like C_3_F_8_ thinking that a prolonged tamponade effect may lead to a larger anatomic success rate [26, 27]. Comparative studies reported similar closure rates in either room air versus SF_6_ and SF_6_ versus C3F8 [28–30]. Also in this study there were no significant differences regarding the anatomic closure, independently of the stage of the macular hole. The repair of the macular hole by ganglion and Muller cells starts from the fourth day after vitrectomy and finishes about on the seventh day and this was demonstrated using OCT observations in research completed by Sato [31] and Masuyama [32]. Our work focused on short term effects of gas tamponade on macular hole recovery and confirmed that the reparation was almost complete at one week after surgery. This provides the rationale for the use of short acting gases or sterile air. C_3_F_8_ provides a prolonged tamponade effect and is usually preferred in case of big size holes. All patients enrolled in this study laid in face-down position for 48 h after surgery. The value of prone position and its duration are still debated. Recent comparative studies reported similar anatomic success rates in face-down positioning and non-supine positioning and comparing long-term and short-term positioning after macular hole surgery [33–37]. Some authors highlight a possible role of strict head position in myopic eyes and in larger holes [35, 38]. A short period of prone position may in the same time promote successful hole closure and reduce patient discomfort as well.

In this study multivariate analysis for repeated measures allowed us to analyse the effect on visual acuity depending on the type of tamponade and to determine the confounding effect on the volume of the hole. All patients experienced a statistically significant improvement of their visual acuity after surgery, but with different velocity, depending on the gas tamponade used. Patients treated with C_3_F_8_ experienced a delay recovery of their BCVA, partly due to the longer time for reabsorption of the tamponade, but reached similar visual results at the last follow up if compared with the ones treated with SF6.

To be able to predict the success of an intervention has always been an arduous challenge for every surgeon. Regarding macular hole repair, several prognostic factors have been investigated, among which duration of the symptoms, stage and dimension of the hole (less or more than 400 microns), preoperative visual acuity. The smaller is the size of the hole, the lower is the time between the onset of symptoms and the intervention, the better are the results, both anatomical and functional. The major diameter of 400 microns may be a valid but approximate cut off to differentiate the macular hole morphology.

In our opinion macular hole volume calculation may be a promising tool to better represent the variable morphology of the holes. To our knowledge no other studies have considered the hole volume as a prognostic tool. Although at present the calculation of the volume requires the caliper tool and may be a laborious task, a tool for automated calculation could be included in the software of the OCT instrument in order to make the procedure quicker. In other papers, minimum hole diameter [39], basal hole diameter [40] and hole height [41] have been studied as prognostic tools for postoperative visual outcomes. We found a correlation between the initial minor diameter and the volume of the hole and the rate of anatomic closure. However further studies are required to confirm these findings.

## Conclusion

In conclusion both SF_6_ and C_3_F_8_ are a valid choice for macular hole surgical repair. Short acting gas has the advantage of involve a faster recovery of visual acuity and a more rapid return to work. This may be useful for those who need to travel in airplane. The initial volume and minor diameter of the hole may be considered as valid tools for predicting surgical success in macular hole repair. The effects of age and gender do not appear to influence the prognosis.

## Abbreviations

SF_6_: sulfur hexafluoride
C_3_F_8_: perfluoropropane
MH: macular hole
IMH: idiopathic macular hole
ILM: internal limiting membrane
OCT: optical coherence tomography
IVTS: international vitreomacular traction study
VMI: vitreomacular interface
PPV: pars plana vitrectomy
BCVA: best corrected visual acuity
V: volume
d: minor diameter
D: major diameter
H: height
